# Activation of the Ahr–IL-6 Axis by Kynurenic Acid Promotes Bone Marrow-Derived MSC Expansion

**DOI:** 10.3390/cimb48010048

**Published:** 2025-12-30

**Authors:** Chi Hung Nguyen, Hang Thi Thu Hoang, Tien Thi Vu, An Dang Pham, Thanh Trung Tran, Taisuke Nakahama, Nam Trung Nguyen

**Affiliations:** 1 Institute of Biology, Vietnam Academy of Science and Technology, 18 Hoang Quoc Viet, Hanoi 10000, Vietnam; nguyenhungchi@gmail.com (C.H.N.); vutien206@gmail.com (T.T.V.); anphamdang92@gmail.com (A.D.P.); trantrungthanh@ibt.ac.vn (T.T.T.); 2 Graduate University of Science and Technology, Vietnam Academy of Science and Technology, 18 Hoang Quoc Viet, Hanoi 10000, Vietnam; 3 Department of RNA Biology and Neuroscience, Osaka University, Osaka 565-0871, Japan; nakahama@rna.med.osaka-u.ac.jp

**Keywords:** kynurenic acid, aryl hydrocarbon receptor, IL-6, mesenchymal stem cell expansion, regenerative medicine

## Abstract

Kynurenic acid (KYNA), a small molecule derived from the tryptophan–kynurenine pathway, can readily diffuse across biological membranes and act as an endogenous ligand for receptors such as the aryl hydrocarbon receptor (Ahr). While KYNA dysregulation is implicated in neurodegenerative disorders, the role of the KYNA–Ahr-IL-6 axis in MSC proliferation and differentiation remains poorly defined. We investigated the impact of KYNA on murine bone marrow-derived MSCs (BM-MSCs) at various concentrations (10–200 μM) and time points (8–48 h). The BM-MSC phenotype was assessed via flow cytometry; proliferation, via cell counting; and the gene expression of *Ahr*, *Cyp1a1*, *Cyp1b1*, and *Il-6,* via quantitative real-time PCR. Multipotency was evaluated through adipogenic, osteogenic, and chondrogenic differentiation assays with histochemical confirmation. KYNA significantly upregulated Ahr mRNA expression. Among the tested concentrations, 100 μM KYNA induced the highest *Ahr* expression (~19.1 ± 1.5-fold greater than that of the untreated controls, *p* < 0.005). Notably, 10 and 50 μM KYNA caused moderate induction, whereas compared with 100 μM KYNA, 200 μM did not further increase expression. In addition, KYN treatment increased *Cyp1a1*, *Cyp1b1*, and *Il-6* expression, with increases of ~64.6 ± 4.5-fold, ~43.6 ± 2.3-fold, and ~41.6 ± 1.2-fold, respectively. Compared with no treatment, 100 µM KYNA enhanced BM-MSC proliferation by 1.210 ± 0.02, 1.189 ± 0.03, and 1.242 ± 0.02-fold across passages P3, P4, and P5, respectively (*p* < 0.05), without altering Sca-1, CD90, or CD45 expression or impairing trilineage differentiation potential. KYNA may activate the AHR–IL-6 signaling axis to promote BM-MSC expansion. This controlled proliferative effect, without loss of phenotypic or functional integrity, highlights the pharmacological potential of KYNA as a small-molecule modulator for stem cell-based therapies.

## 1. Introduction

Mesenchymal stem cells (MSCs) are multipotent adult stem cells found in diverse tissues, including the bone marrow, umbilical cord, adipose tissue, and placenta [1]. They are considered promising therapeutic tools for immune-mediated and inflammatory disorders because of their ability to modulate immune responses and exert anti-inflammatory effects via multiple signaling pathways [2]. MSCs influence both adaptive and innate immunity, including by suppressing T-cell proliferation and cytokine secretion; promoting regulatory T-cell generation; regulating B-cell proliferation and differentiation; and modulating monocytes, macrophages, dendritic cells, and natural killer (NK) cells, partly through the secretion of inflammatory cytokines such as interleukin-6 (IL-6) [3,4,5].

Kynurenic acid (KYNA) is an endogenous metabolite of the essential amino acid tryptophan and is generated via the kynurenine (KYN) pathway through the enzymatic action of indoleamine-2,3-dioxygenase (IDO) [6]. More recently, KYNA has emerged as an immunomodulatory molecule that influences immune regulation and tissue homeostasis [7]. Both KYN and KYNA act as endogenous ligands for the aryl hydrocarbon receptor (Ahr), a ligand-activated transcription factor involved in xenobiotic metabolism, immune regulation, cell proliferation, and differentiation [8].

Our group and others have demonstrated that Ahr activation by KYN can modulate the production of cytokines and influence the immunoregulatory properties of multiple cell types, including dendritic cells and T cells [9,10,11]. Given the known roles of IL-6 in MSC proliferation, the interaction between KYNA and the Ahr–IL-6 axis represents a compelling mechanism to explore. IL-6 is a powerful cytokine that acts as a proinflammatory mediator, playing a central role in inflammatory diseases [12]. In addition, the cytochrome P450 (CYP) enzymes Cyp1a1 and Cyp1b1, which are downstream products of Ahr activation, play critical roles in carcinogenesis [13]. Furthermore, several compounds, such as nanocurcumin, have recently been shown to stimulate the proliferation of MSCs [14]. Taken together, these observations suggest that the function of KYNA in immune regulation may extend to the control of MSC biology. In particular, identifying how KYNA affects bone marrow-derived MSC (BM-MSC) proliferation and differentiation could reveal new strategies to increase BM-MSC expansion and therapeutic efficacy in regenerative medicine and the treatment of immune-related diseases.

## 2. Materials and Methods

### 2.1. BM-MSC Culture and KYNA Treatment

Bone marrow-derived mesenchymal stem cells (BM-MSCs) were isolated from the femurs and tibias of 6-week-old Swiss mice (n = 5 per experiment). All experimental protocols were approved by the Scientific Council of Institute of Biology, VAST, Vietnam. The bone marrow cells were flushed out and seeded into culture wells. After 24 h of incubation, the BM-MSCs had adhered to the bottom of the wells, whereas the nonadherent cells remained suspended. The culture medium was then removed, and the wells were gently washed with phosphate-buffered saline (PBS). The adherent BM-MSCs were further cultured under standard conditions for approximately 10 days, during which they reached approximately 80% confluence. At this point, the BM-MSCs were harvested and passaged as needed. After 10 days of culture, BM-MSCs at passage 0 (P0) were collected, with a yield of approximately 5 × 10^5^ cells per mouse, and were isolated from the femur and tibia bone marrow of each mouse. MSCs from passages P1 to P5 were seeded at a density of 5 × 10^4^ cells/cm^2^ and cultured in DMEM supplemented with 10% fetal bovine serum (FBS), either in the presence or absence of 10–200 μM KYNA (Thermo Fisher Scientific, Fair Lawn, NJ, USA), as previously described [15].

### 2.2. Flow Cytometry

At P4, the cells were detached from the cultured disks via 0.25% trypsin (Gibco). Then, the cells were fixed in 4% formaldehyde for 15–20 min at room temperature, blocked in staining buffer (PBS, 0.5% BSA, 2 mM EDTA), and incubated with antibodies against Sca-1, CD90, and CD45 (Miltenyi Biotec) at a 1:50 dilution (or a final concentration of 10 µg/mL), as specified in Supplementary Table S1. Flow cytometry was performed with a MACS Quant VYB system and software (Miltenyi Biotec, version 3.0.1).

### 2.3. Differentiation Assay

BM-MSCs were seeded into 24-well culture plates for lineage-specific differentiation. For adipogenic induction, the cells were plated at a density of 1.5 × 10^4^ cells/cm^2^ and cultured until they reached ~90% confluence. Differentiation was initiated via the MesenCult™ Adipogenic Differentiation Kit (Mouse; STEMCELL Technologies, Vancouver, BC, Canada), with the medium changed every 3–4 days over a 7-day period. Lipid accumulation was assessed by Oil Red O staining following fixation with 4% paraformaldehyde. For osteogenic differentiation, cells were seeded at 5 × 10^4^ cells/cm^2^ and induced at ~90% confluence with the MesenCult™ Osteogenic Stimulatory Kit (Mouse; STEMCELL Technologies, Vancouver, BC, Canada) for 14 days. Cultures were maintained with regular medium replacement, and calcium deposition was visualized by Alizarin Red S staining after fixation. Chondrogenic differentiation was performed via the pellet culture method. Briefly, 1 × 10^5^ BM-MSCs were placed in 15 mL polypropylene tubes and centrifuged at 300× *g* for 5–10 min to form a cell aggregate. The pellets were incubated under standard culture conditions (37 °C, 5% CO_2_) in MesenCult™-ACF Chondrogenic Differentiation medium (STEMCELL Technologies, Vancouver, BC, Canada) for 21 days. The medium was changed every 2–3 days without disturbing the pellet. At the end of the induction period, the pellets were fixed in 10% neutral buffered formalin, embedded in paraffin, and sectioned at 6 µm. The sections were stained with Alcian blue to detect sulfated proteoglycans, counterstained with Nuclear Fast Red, and examined via light microscopy.

### 2.4. Quantitative Real-Time Polymerase Chain Reaction (PCR)

At P4, total RNA was isolated from BM-MSCs via TRIzol reagent (Thermo Fisher Scientific, Carlsbad, CA, USA) according to the manufacturer’s instructions. A total of 1 μg of RNA was converted into cDNA via the RevertAid First Strand cDNA Synthesis Kit (Thermo Fisher Scientific, CA, USA). Relative gene expression was measured via PowerUp SYBR Green Master Mix (Thermo Fisher Scientific, USA). Primers were designed by Primer3 web interface (v4.1.0), and the sequences were as follows (5′-3): *Ahr*-F: GACAGTTTTCCGGCTTCTTG; *Ahr*-R: CGCTTCTGTAAATGCTCTCG; *Cyp1a1*-F: CCCACAGCACCACAAGAGATAC; *Cyp1a1*-R: CTTGCCCAAACCAAAGAGAGTGAC; *Cyp1b1*-F: CCACTATTACGGACATCTTCGG; *Cyp1b1*-R: CACAACCTGGTCCAACTCAG; *Il-6*-F: TACTTCACAAGTCCGGAGAGG; *Il-6*-R: TCCACGATTTCCCAGAGAAC; *β*-*actin*-F: GCTCTTTTCCAGCCTTCCTTC; *β*-*actin*-R: GGTGCTAGGAGCCAGAGCAG [16,17]. The qRT-PCRs and analyses were performed via the QuantStudio™ 6 Pro Real-Time PCR System with Design & Analysis Software v2.6.0. The relative expression levels of the genes were calculated via the 2^∆∆Ct^ method [18]. The β-actin gene was used as an endogenous control to normalize gene expression levels. The graphs and data were processed via Microsoft Excel, with the *p* value via the t test method.

### 2.5. Cell Counting Assay

A trypan blue exclusion assay was used to determine the total number of BM-MSCs treated with or without KYNA. A 10 µL aliquot of the cell suspension was then mixed with 10 µL of 0.4% trypan blue solution for staining. Subsequently, 10 µL of the stained mixture was loaded onto a hemocytometer for cell counting under a light microscope [19].

### 2.6. Data Analysis

The results are expressed as the means ± standard deviations (SDs). Statistical analysis was performed via Student’s *t* test or ANOVA as appropriate, with *p* values less than 0.05 considered significant.

## 3. Results

### 3.1. KYNA Upregulates Ahr Expression in BM-MSCs

**Figure 1:**
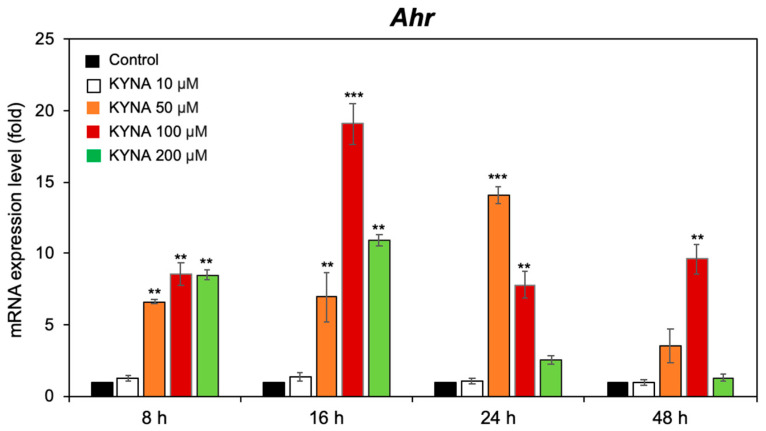
KYNA significantly increases the upregulation of *Ahr* expression in BM-MSCs. BM-MSCs were treated with 10, 50, 100, or 200 μM KYNA for 8, 16, 24, or 48 h, and *Ahr* mRNA expression was quantified via qPCR. The data are expressed as the means ± SDs from three independent experiments, with values normalized to those of untreated controls at each time point. ** *p* < 0.01, *** *p* < 0.005 vs. the untreated control.

To investigate whether KYNA modulates *Ahr* expression in BM-MSCs, cells were exposed to increasing concentrations of KYNA (10, 50, 100, and 200 μM) for 8, 16, 24, or 48 h. qPCR analysis revealed a significant increase in *Ahr* mRNA (Figure 1). At 8 h, moderate upregulation was detected only at concentrations ≥ 50 µM. By 16 h, a further increase in *Ahr* expression was observed, with the greatest increase (~19.1 ± 1.5-fold vs. the control) observed at 100 µM. This elevated expression was sustained at 24 h and 48 h in the 100 µM group. At 24 h, all the concentrations again significantly increased, peaking at ~14.1 ± 0.6-fold greater than that of the control in the 50 µM group. In contrast, 10 µM KYNA produced no significant effect, and 200 µM KYNA failed to maintain high expression at 48 h. On the basis of these results, 100 µM KYNA was selected for subsequent MSC experiments. These findings indicate that KYNA robustly activates *Ahr* transcription in BM-MSCs, with both the concentration and exposure duration influencing the magnitude of the response.

### 3.2. KYNA Does Not Influence BM-MSC Morphology or Phenotype

**Figure 2:**
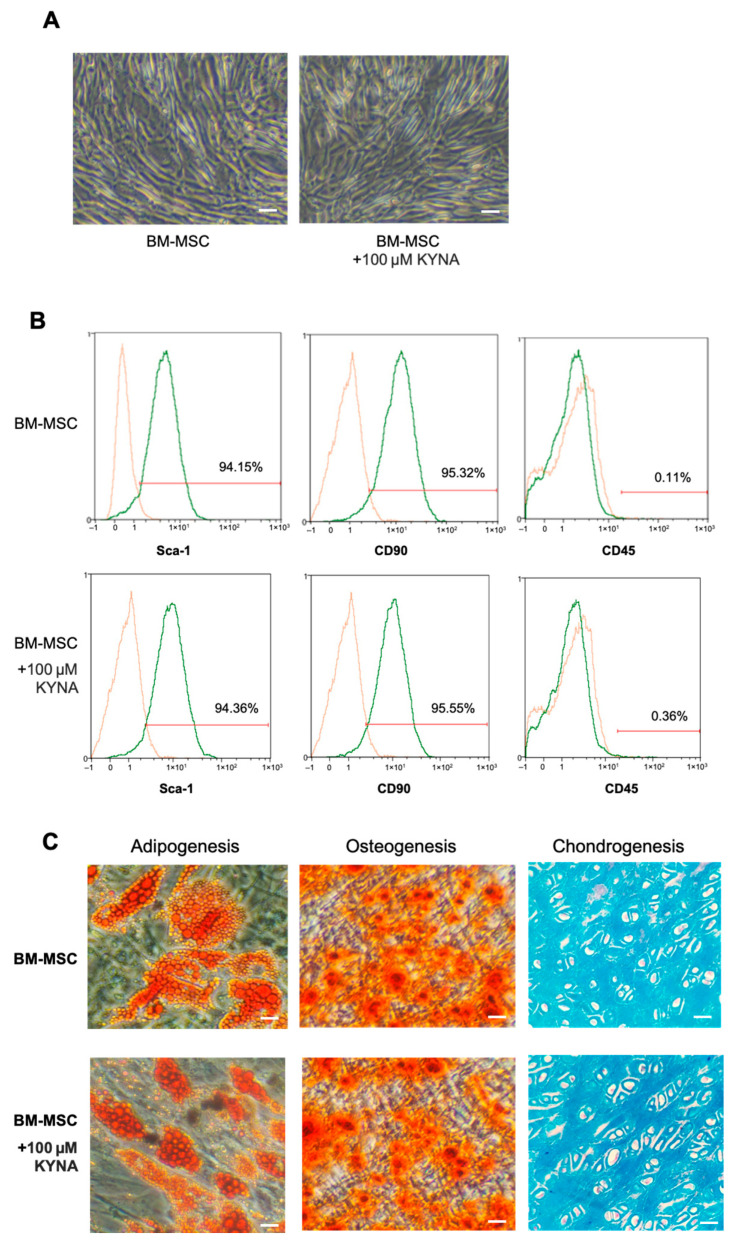
KYNA does not alter BM-MSC morphology, phenotype, or multipotent differentiation potential. (**A**) Phase-contrast microscopy images of BM-MSCs at P4 after 7 days of culture in complete medium with or without 100 μM KYNA showing no detectable changes in fibroblast-like morphology (magnification, 4×). Scale bar: 50 μm (**B**) Flow cytometry analysis of BM-MSC surface markers after 7 days of culture with or without 100 μM KYNA confirmed a typical MSC phenotype: positive for Sca-1 and CD90 and negative for the hematopoietic marker CD45. The panel of cell surface markers consisted of anti-Sca-1, anti-CD90, and anti-CD45 antibodies. The orange histogram represents control cells, and the green histogram represents the cells incubated with the indicated antibodies. (**C**) Representative images of adipogenic, osteogenic, and chondrogenic differentiation after 14 days (adipogenesis and osteogenesis) or 21 days (chondrogenesis) of induction, showing no apparent differences between KYNA-treated and untreated BM-MSCs. Red curves represent the positive cells.

To determine whether KYNA affects the basic characteristics of BM-MSCs, we examined their morphology, surface marker expression, and multipotent differentiation potential. Phase-contrast microscopy revealed that BM-MSCs at P4 maintained their characteristic fibroblast-like morphology after 7 days of culture in complete medium with or without KYNA at a concentration of 100 μM (Figure 2A). Flow cytometry analysis confirmed that KYNA-treated BM-MSCs retained their typical immunophenotype, being positive for Sca-1 and CD90 and negative for the hematopoietic marker CD45 (Figure 2B and Table 1). Furthermore, KYNA treatment (100 μM) did not alter the adipogenic, osteogenic, or chondrogenic differentiation capacity of BM-MSCs, as shown by Oil Red O, Alizarin Red S, and Alcian blue staining, respectively (Figure 2C). These results indicate that KYNA does not affect the morphology, surface marker expression, or multipotency of BM-MSCs under the tested conditions.

### 3.3. KYNA Increases Ahr-Related Cyp1a1, Cyp1b1, and Il-6 Expression in BM-MSCs

**Figure 3:**
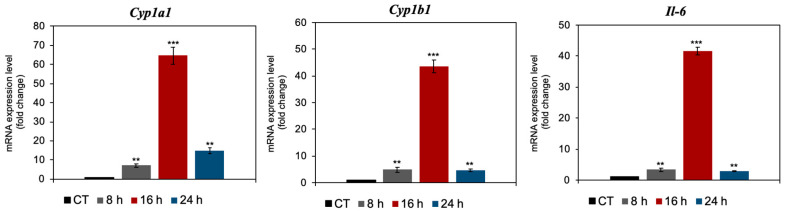
KYNA induces the expression of *Cyp1a1*, *Cyp1b1*, and *Il-6* in BM-MSCs. BM-MSCs were treated with 100 μM KYNA or the control (CT) for 8, 16, or 24 h, and gene expression was quantified via qPCR. The data represent the means ± SDs from three independent experiments, with values normalized to those of untreated controls. ** *p* < 0.01, *** *p* < 0.005 vs. the untreated control.

To assess the downstream transcriptional effects of AHR activation, we examined the expression of *Cyp1a1*, *Cyp1b1*, and *Il-6* in BM-MSCs following treatment with 100 μM KYNA for 8, 16, and 24 h. qPCR analysis revealed a marked time-dependent induction of all three genes compared with those in untreated controls. *Cyp1a1* and *Cyp1b1* expression peaked at 16 h, increasing ~64.6 ± 4.5-fold and ~43.6 ± 2.3-fold, respectively, before decreasing at 24 h. Similarly, *Il-6* expression increased sharply at 16 h (~41.6 ± 1.2-fold), followed by a sharp reduction at the later time points (Figure 3). These results confirm that KYNA robustly activates canonical AHR target genes and proinflammatory cytokine expression in BM-MSCs, with maximal induction occurring at 16 h.

### 3.4. KYNA Promotes BM-MSC Proliferation

**Figure 4:**
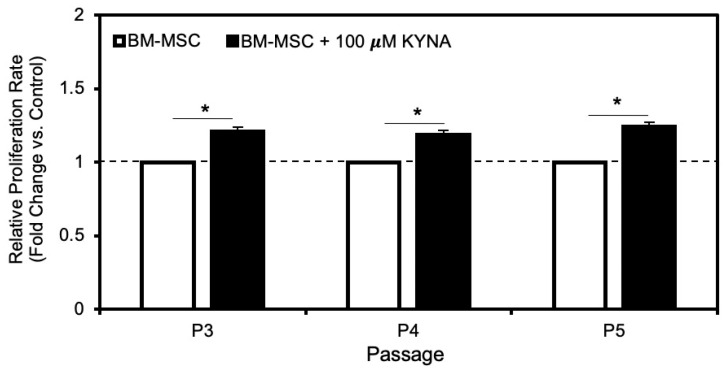
Effect of 100 µM KYNA on the proliferation of BM-MSCs at different passages. BM-MSCs at P3, P4, and P5 were cultured with or without 100 µM KYNA. The proliferation rates of KYNA-treated cells were compared to those of untreated controls at all tested passages. The data are presented as the means ± SDs from triplicate experiments. * *p* < 0.05 versus the corresponding untreated control—Control Baseline.

At passages P3, P4, and P5, the proliferation rates of BM-MSCs cultured with 100 μM KYNA were 1.210 ± 0.02, 1.189 ± 0.03, and 1.242 ± 0.02-fold greater than those of their respective untreated controls. Overall, KYNA treatment significantly enhanced BM-MSC proliferation by an average of approximately 1.2-fold across passages (Figure 4).

## 4. Discussion

KYNA induced Ahr expression in BM-MSCs at 100 µM, resulting in the most robust and sustained activation, peaking at 16 h and persisting, although it was attenuated at 24 and 48 h (Figure 2). Neither the low dose (10 µM) nor the high dose (200 µM) generated a comparable sustained effect. This biphasic pattern aligns with the known sensitivity of Ahr signaling to ligand concentration and its negative feedback regulation via the Ahr repressor (Ahrr) or ligand metabolism by Cyp1a1 [20,21].

This study examined whether KYNA, an endogenous tryptophan metabolite and AHR ligand, modulates the fundamental properties of murine BM-MSCs, with particular attention given to morphology, phenotype, Ahr signaling, and proliferation. After 7 days of culture with KYNA (10–200 µM), BM-MSCs retained their typical fibroblast-like morphology and characteristic surface marker profile—positive for Sca-1 and CD90 and negative for CD45—indicating that KYNA does not compromise their phenotypic identity or stemness (Figure 3). Maintaining these traits is critical for MSC therapeutic applications, as phenotypic shifts often reflect differentiation, senescence, or loss of multipotency [22,23].

Consistent with canonical Ahr activation, KYNA markedly upregulated the classical target genes *Cyp1a1*, *Cyp1b1*, and *Il-6* (Figure 4). Both *Cyp* genes peaked at 16 h (64.6 ± 4.5-fold and ~43.6 ± 2.3-fold increases, respectively), mirroring the *Ahr* activation profile. *Il-6* expression follows a similar kinetic pattern, which is consistent with prior reports that Ahr ligands can drive *Il-6* transcription, influencing both pro- and anti-inflammatory pathways depending on the cellular context [24]. The modulation of the expression of the *Cyp1a1*, *Cyp1b1*, and *Il-6* genes represents an area of profound interest with significant therapeutic implications. The results obtained, which demonstrate the specific regulation of these biomarkers, not only contribute to the understanding of the underlying molecular pathways but also open the door to their direct application in the development of more effective clinical strategies.

**Figure 5:**
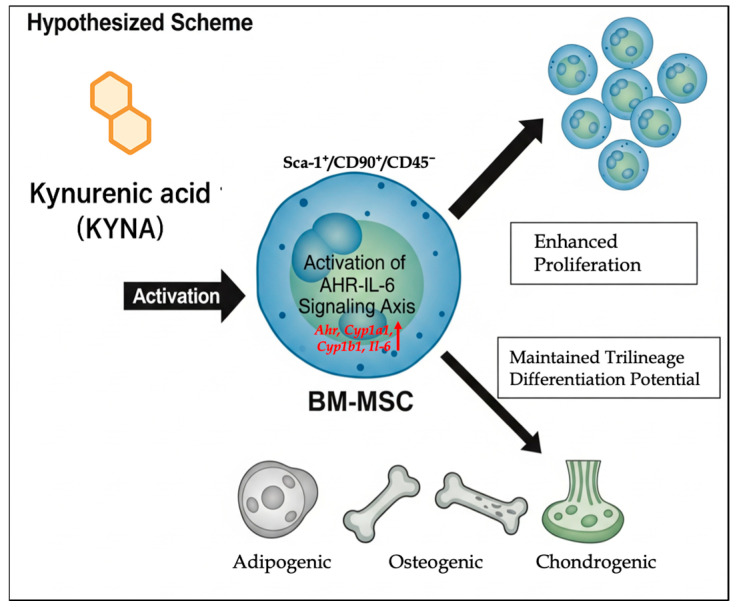
Schematic illustration of the effects of KYNA on murine BM-MSCs. KYNA, an endogenous metabolite of the tryptophan–IDO pathway, binds and activates *Ahr*. This activation upregulates the downstream target genes *Cyp1a1*, *Cyp1b1* and *Il-6*. The combined transcriptional changes enhance BM-MSC proliferation by ~1.2-fold relative to that of untreated controls while maintaining the MSC phenotype (Sca-1^+^/CD90^+^/CD45^−^) and preserving multipotency toward adipogenic, osteogenic, and chondrogenic lineages.

Functionally, 100 µM KYNA enhanced BM-MSC proliferation by ~1.2-fold across passages P3–P5 (Figure 5), suggesting a sustained proliferative stimulus even during later culture stages, when MSC growth typically decreases due to senescence [25,26]. The combination of modest, transient Ahr activation and IL-6 induction may underlie this effect, as IL-6 has been implicated in MSC proliferation and immunomodulation [27,28]. Compared with high-affinity exogenous ligands such as TCDD, the relatively weak agonist KYNA may support proliferation without triggering differentiation or loss of stemness (Figure 5).

Limitations of this work should be noted. While this study rigorously details the molecular mechanism and functional outcome of KYNA in promoting proliferation while maintaining the multipotency of murine bone marrow derived MSCs (BM-MSCs), the restriction to a single species and tissue source is a primary limitation for clinical translation. The properties of mesenchymal stem cells, including their proliferation rate and differentiation potential, are known to be dependent on their tissue of origin, a concept often linked to epigenetic memory. Therefore, future investigations are critically warranted to assess the pharmacological effects of KYNA on MSCs derived from other clinically relevant sources, such as adipose derived MSCs (AD-MSCs), which are known for their strong proliferative capacity, and umbilical cord derived MSCs (UC-MSCs), to definitively confirm the generalizability and full translational potential of KYNA as a small-molecule additive for clinical-grade cell expansion.

Taken together, these findings highlight KYNA as a promising, naturally occurring modulator of BM-MSC expansion that preserves key stem cell properties. By activating the Ahr–IL-6 axis without inducing differentiation or senescence, KYNA may help overcome one of the central challenges in regenerative medicine—the need for robust, scalable MSC expansion while maintaining functional potency. This approach could be applied to optimize MSC manufacturing for cell-based therapies for tissue repair, immune modulation, and inflammatory disease treatment.

## 5. Conclusions

This study demonstrated that KYNA enhances the proliferation of murine BM-MSCs by approximately 1.2-fold compared with that of controls without altering their morphology, surface marker profile, or multipotent differentiation capacity. KYNA treatment upregulated the expression of *Ahr*, *Cyp1a1*, *Cyp1b1*, and *Il-6*, suggesting that activation of the Ahr–IL-6 signaling axis is a potential mechanism underlying its proliferative effect. The specific regulation of *Cyp1a1*, *Cyp1b1*, and *Il-6* gene expression has significant therapeutic implications, primarily in oncology and inflammatory/autoimmune diseases. These genes serve as crucial biomarkers and therapeutic targets for developing more effective and personalized clinical strategies. The ability of KYNA to stimulate BM-MSC expansion while preserving stemness highlights its potential utility for BM-MSC amplification in regenerative medicine and cell-based therapies.

## Figures and Tables

**Table 1:** Expression of BM-MSC surface markers.

Marker	BM-MSC (%) *	BM-MSC + 100 µM KYNA (%) *	*p* Values
Sca-1	94.5 ± 0.3	95.0 ± 0.5	>0.05
CD90	95.6 ± 0.4	95.5 ± 0.3	>0.05
CD45	0.12 ± 0.03	0.33 ± 0.04	>0.05

* Values represent the means ± SDs of three independent experiments.

## Data Availability

The datasets used and/or analyzed during the current study are available from the corresponding author upon reasonable request.

## Supplementary Materials

The following supporting information can be downloaded at https://www.mdpi.com/article/10.3390/cimb48010048/s1.

## Abbreviations

The following abbreviations are used in this manuscript:

BM-MSC	Bone marrow derived Mesenchymal stem cells
DMEM	Dulbecco′s Modified Eagle′s Medium
AHR	Aryl hydrocarbon receptor
IDO	Indoleamine 2,3-dioxygenase
KYNA	Kynurenic acid
IL-6	Interleukin-6
CYP1A1	Cytochrome P450 family 1 subfamily A member 1
CYP1B1	Cytochrome P450 family 1 subfamily B member 1
Sca-1	Stem cells antigen-1
