# Supplementary material for: Activation of the Ahr–IL-6 Axis by Kynurenic Acid Promotes Bone Marrow-Derived MSC Expansion
Source: Curr Issues Mol Biol. 2025 Dec 30;48(1):48. doi: 10.3390/cimb48010048 (PMC12839728; doi:10.3390/cimb48010048)
Supplement: Supplementary file 1 [file cimb-48-00048-s001.zip › cimb-4018805-supplementary.pdf]

Supplementary Table S1: Flow Cytometry Reagent Specifications

Antibody Target	Manufacturer	Catalogue Number	Dilution / Final Concentration	Conjugated (Yes/No)	Isotype Control
Sca-1	Miltenyi Biotec	130-106-259	1:50	Yes	REA Control Antibody
CD90	Miltenyi Biotec	130-128-976	1:50	Yes	REA Control Antibody
CD45	Miltenyi Biotec	130-123-900	1:50	Yes	Isotype Control Antibody, rat IgG2b
